# Patient’s perspective of sustained remission in rheumatoid arthritis

**DOI:** 10.1186/s12891-017-1717-8

**Published:** 2017-09-02

**Authors:** Irazú Contreras-Yáñez, Guillermo Guaracha-Basañez, Daniel Ruiz-Domínguez, Virginia Pascual-Ramos

**Affiliations:** 1 Department of Immunology and Rheumatology, Instituto Nacional de Ciencias Médicas y Nutrición Salvador Zubirán, Vasco de Quiroga 15, Colonia Belisario Domínguez, Sección XVI, 14080 México City, CP Mexico; 2 Department of Internal Medicine, Instituto Nacional de Ciencias Médicas y Nutrición Salvador Zubirán, Vasco de Quiroga 15, Colonia Belisario Domínguez, Sección XVI, 14080 México City, CP Mexico

**Keywords:** Patient-reported-outcomes, Remission, Rheumatoid arthritis

## Abstract

**Background:**

During the course of rheumatoid arthritis (RA), patients have profound negative effects on their patient-reported-outcomes (PRO); in addition, the impact of sustained remission (SR) on PROs may differ for each particular outcome. The objectives of this study were to identify SR from an inception cohort of RA patients and to examine the impact of SR in an ample spectrum of PROs.

**Methods:**

The study was developed in a well characterized and ongoing cohort of RA patients with recent onset disease recruited from 2004 onwards. In November 2016, the cohort included 187 patients, of whom 145 had at least 30 months of follow-up, with complete rheumatic assessments at regular intervals in addition to a pain visual analogue scale (PVAS), overall disease-VAS (OVAS), health assessment questionnaire (HAQ), Short-Form 36v2 Survey (SF-36) and fatigue assessment. First SR was defined according to the DAS28 cut-offs (DAS28-SR) and ACR/EULAR 2011 Boolean definition (B-SR), if maintained for at least 12 consecutive months. The dependent t test and Mc Nemar’s tests were used for comparisons between related groups. Local IRB approval was obtained.

**Results:**

More patients achieved DAS28-SR than B-SR: 78 vs. 63, respectively. Fifty patients met both SR definitions. Follow-up to DAS28-SR was shorter than to B-SR and the duration of DAS28-SR was longer, *p* ≤ 0.023 for all comparisons.

At SR, patients had PRO proxy to normal values; the percentage of patients with normal PRO varied from 97% (95% CI: 91–99) for HAQ to 50% (95% CI: 39–61) for absence of fatigue.

In DAS28-SR patients, acute reactant phases within the normal range were detected very early (after 1.5–2.9 months). HAQ, PVAS, OVAS and SF-36 were scored within the normal range after 6–7 months. The absence of fatigue was detected at 8.7 months of follow-up, which was similar to DAS28-SR. In the 63 patients with B-SR, a similar pattern was observed. The follow-up to outcomes of the 50 patients who met both SR definitions was similar, but the absence of fatigue and physical component SF-36 normalization were achieved earlier in B-SR patients (*p* ≤ 0.02).

**Conclusions:**

The impact of SR on PRO differs accordingly to each particular outcome.

## Background

Clinical remission has become a widely accepted treatment goal in rheumatoid arthritis (RA) patients with early disease. Reaching remission, however, is only the first step in a targeted treatment approach, and sustained remission (SR) is the desirable subsequent step. Previous studies have demonstrated that the progression of joint damage decreases with increasing duration of remission [1]; nonetheless, the ultimate target to drive treatment decisions in RA patients should be better long-term patient-reported outcomes [2].

In the last few decades, international efforts had been committed to defining remission in RA. Various definitions are available, with different levels of stringency [3–5]. Ultimately, in RA patients, remission may be operationalized as either a complete absence of disease activity or a level of disease activity that is so low that it is not troublesome to the patient and portends a good prognosis [6]. This definition is particularly relevant given that during the course of RA, affected individuals face considerable physical, psychological and social changes that have profound negative effects on their health-related QoL (HRQoL) [7]. However, when remission is achieved, RA patients also achieved population norms in terms of HRQoL, unlike in the case of patients with other rheumatic diseases, such as patients with ANCA-associated vasculitides in remission, who exhibit substantially reduced patient-reported outcomes (PRO) [8].

Current RA management guidelines recommend incorporating patient-reported measures of functioning and quality of life into clinical trials as they are as effective as traditional physician- or laboratory-reported outcomes in reflecting long-term morbidity and mortality, easier to administer and less expensive than physician-observed health status measures [9–11]. Moreover, clinicians and patients have different perspectives, and patients prioritize clinical outcomes that are not routinely measured [10, 12]. PROs include outcomes that are limited to a symptom (such as pain or fatigue) or those that evaluate complex constructs, such as patient’s function, disability or the overall disease status. One relevant aspect of the impact of SR on PROs is that it may differ for each particular outcome. In the present study, we sought to identify SR patients from an inception and ongoing cohort of RA patients with recent onset disease at cohort enrollment and examine the impact of SR in an ample spectrum of PROs.

The specific objectives were as follows:

- To describe PROs from RA patients who achieved SR for the first time as well as the proportion of those patients who achieved PRO norms according to 2 definitions of SR.
- To describe the time to normalization of PROs in RA patients who achieved SR.

## Methods

### The early RA cohort

In February 2004, an ongoing cohort of patients with recent-onset RA (within 12 months of symptoms onset) was initiated. At inclusion, the complete medical history and sociodemographic data were recorded in addition to the type(s) and levels of rheumatoid factor (RF) and antibodies against cyclic citrullinated proteins (ACCP). Consecutive medical evaluations were standardized and scheduled at regular intervals: 2 months apart for the first 2 years of follow-up and then 2, 4 or 6 months apart depending on patient and disease characteristics. When patients required additional consultations because adverse events or unexpected conditions (pregnancy, flares, comorbidity, etc.…) a visit with the attending rheumatologist was scheduled within 1 week.

Clinical assessments always included at least swollen and tender joint counts, physician overall disease activity, erythrocyte sedimentation rate (ESR) and C-reactive protein (CRP) level, comorbidities (established by a record review) and Charlson score [13], and treatment assessments. In addition, the following PROs were evaluated before clinical assessments that were performed by a single rheumatologist: a pain visual analogue scale (P-VAS), overall disease-VAS (O-VAS), health assessment questionnaire (HAQ) [14], Short-Form 36v2 Survey (SF-36) [15, 16] and (presence/absence) of fatigue.

Patients had health expenditures government coverage depending on their incomes but needed to pay for their medication and these were not provided by the local pharmacy (unless patients were hospitalized and if available). Treatment was prescribed by the rheumatologist in charge of the clinic, was “Treat-to-target” oriented and according to the current standard of care (no protocol was followed). Traditional Disease Modifying Anti-Rheumatic Drugs (DMARDs) were used in 98% of them with/without corticosteroids (50% of patients received low doses of oral corticosteroids during their follow-up). Subcutaneous methotrexate was the first DMARD indicated (unless contraindicated) although most of the patients (72%) had 2–3 combined DMARDs during their follow-up [17]. When flares were identified, corticosteroids and/or DMARDs were added or doses increased (if suboptimal). Patient’s preferences and resources were always considered.

In November 2016, the cohort comprised 187 RA patients with a variable follow-up recruited from 2004 onwards. Of these patients, 145 had at least 30 months of follow-up (baseline and consecutive visits numbered as 2 to 16), which was deemed to be convenient to accomplish the objectives described, including achieving a SR state. We previously reported that patients from the Early Arthritis Clinic (EAC) achieved their first sustained remission state at 14 ± 9 months of follow-up [18].

### Definitions

*Sustained remission (SR)* was defined according to disease activity score on 28 joints (DAS28) cut-offs (DAS28-SR) and according to the American College of Rheumatology (ACR)/ European League Against Rheumatism (EULAR) 2011 Boolean definition (B-SR).

*First DAS28-SR* was considered (Yes/No) to occur when patients achieved at least 12 months of continuous follow-up with DAS28 < 2.4 for the first time [3, 19].

*First B-SR* was considered (Yes/No) to occur when patients achieved at least 12 months of continuous follow-up including swollen a 28-joint count ≤1 AND tender 28-joint count ≤1 AND CRP ≤ 1 mg/dL AND patient O-VAS ≤1 (0–10 cm) for the first time [4].

*Time in SR* was computed from the first visit (time) that SR was achieved up to the last follow-up, with SR according to the definition used.

*HAQ norm* (HAQ-N) was considered if ≤0.25 on a scale from 0 to 3 [20].

*SF-36 norm* (SF-36-N, global score and either mental or physical scores) was considered if ≥80 on a scale from 0 to 100. The cut-off was derived from data obtained from a healthy Mexican population [21].

*PVAS and OVAS norms* (PVAS-N and OVAS-N) were defined as ≤10 on a scale from 0 to 100 mm [22].

*Substantial fatigue (presence/absence)* was defined after re-scoring the vitality domain of the SF-36v2 domain. Data are presented as a continuous variable (on a 0 to 100 scale) and a dichotomus variable (absence/presence). Substantial fatigue was arbitrarily defined if the mean of the four items included in the vitality domain was <80.

The cut-offs for ESR (Westergren method) were <30 mm/H for females or <20 mm/H for males [5].

The cut-off for CRP (Nephelometry) was defined as ≤1.57 mg/dL according to manufacturer’s recommendations (Beckman Coulter, Inc.).

*Physician overall disease VAS normalization* (PhyVAS-N) was defined as ≤10 mm on a scale from 0 to 100.

PROs during SR were recorded at the same time point: at least 6-months of follow-up with SR before and after PROs were recorded.

### Statistics

Descriptive statistics was used, number and percentage (with 95% CI) for dichotomus variables and (mean ± SD) for continuous variables. The dependent t test and Mc Nemar’s tests were used for comparisons between related groups, either as percentages or the (mean ± SD). Gender has been associated to unfavorable outcomes [23] and analysis were repeated in female and male subpopulations. Relevant results are presented.

All statistical tests were 2-sided and evaluated at the 0.05 significance level. Statistical analysis was performed using the SPSS/PC program (v.17.0; Chicago IL).

### Ethics

The present study was approved by the Institution’s internal review board “Comité de ética del Instituto Nacional de Ciencias Médicas y Nutrición Salvador Zubirán”, with the reference number IRE-274-10/11–1. Written informed consent was obtained from all patients to have their charts reviewed and data presented in scientific forums or published.

## Results

### Characteristics of the entire population and comparison of females and males patients with DAS28-SR and with B-SR.

The entire population comprised 145 RA patients, among whom 130 (89.7% [85–95]) were females; their data are summarized in Table 1 (first column).

**Table 1:** Baseline and cumulative characteristics from the entire population and comparison of females and males patients with DAS28-SR and with B-SR

	Entire population *N* = 145	Females, *N* = 130		Males, *N* = 15		p^1^/p^2^
		DAS28-SR *N* = 68	B-SR *N* = 55	DAS28-SR *N* = 10	B-SR *N* = 8	
Baseline characteristics^a^						
Age, years	37.9 ± 12.9	34.3 ± 11.5	35.6 ± 12.2	36.9 ± 12	45.6 ± 6.3	0.22/**0.01**
Years of formal education	11.1 ± 3.9	12 ± 3.8	11.9 ± 3.7	11.8 ± 3.6	10.6 ± 2.6	0.39/0.29
N° (% [95% CI]) of patients RF+	118 (81.4 [75–88])	52 (76.5 [66–87])	43 (78.2 [67–89])	9 (90 [30–95])	8 (100 [N.A.])	0.45/0.33
N° (% [95% CI]) of patients ACCP+	123 (84.8 [79–91])	54 (79.4 [70–89])	43 (78.2 [67–89])	9 (90 [30–65])	7 (87.5 [25–89])	0.68/1
Symptoms duration, months	5.4 ± 2.6	5.5 ± 2.7	5.4 ± 2.3	5.3 ± 2.2	4.3 ± 1.4	0.39/0.16
DAS28	5.9 ± 1.4	5.6 ± 1.5	5.6 ± 1.5	5.6 ± 1.4	5.5 ± 0.7	0.59/0.58
N° (% [95% CI]) of patients with DMARDs at referral	37 (25.5 [18–33])	17 (25 [15–35])	15 (27.3 [16–39])	4 (40 [10–70])	2 (25 [5–55])	0.27/1
N° (% [95% CI]) of patients with CTs at referral	40 (27.6 [20–35])	22 (32.4 [21–44])	13 (23.6 [12–35])	4 (40 [10–70])	2 (25 [5–55])	0.72/1
N° (% [95% CI]) of patients with comorbidity	60 (41.4 [33–95])	33 (48.5 [37–60])	28 (50.9 [38–64])	7 (70 [10–71])	5 (62.5 [5–65])	0.31/0.71
Charlson score	1.4 ± 0.6	1.5 ± 0.6	1.5 ± 0.6	1.6 ± 0.7	1.8 ± 0.7	0.08/0.26
Cumulative (up to SR) characteristics						
DAS28	Not aplicable	2.3 ± 0.6	2.3 ± 0.6	2.3 ± 0.7	2.5 ± 0.9	0.99/0.59
N° DMARD/patient	1.8 ± 0.7	1.7 ± 0.5	1.7 ± 0.5	1.6 ± 0.6	1.5 ± 0.5	0.4/0.29
N° (% [95% CI]) of patients with CTs	58 (40 [32–48])	28 (41.2 [30–53])	21 (38.2 [25–51])	5 (50 [19–81])	2 (25 [5–55])	0.76/1
N° of comorbidity/patient	1.6 ± 0.8	1.3 ± 0.8	1.5 ± 0.9	1.5 ± 0.8	1.8 ± 0.7	**0.03**/0.26

p^1^ = Comparison of DAS28-SR females and DAS28-SR males

p^2^ = Comparison of B-SR females and B-SR males

CI = Confidence interval. RF = Rheumatoid factor. ACCP = Antibodies to cyclic citrullinated peptides. DAS28 = Disease activity index (28 joints evaluated). DMARDs = Disease modifying anti-rheumatic drugs. CTs = Corticosteroids

^a^Data presented as (mean ± SD) and number (percentage [95% CI])

In the entire population, an increased number of patients achieved DAS28-SR compared to B-SR, 78 vs. 63 patients out of 145, respectively. There were 50 patients who met bot definitions of SR. In those patients, follow-up to DAS28-SR was shorter than to B-SR and the duration of DAS28-SR was longer than the duration of B-SR. Similar results were obtained in the subpopulation of females and a similar tendency was seeing in the subpopulation of males but differences did not showed statistical significance due to the limited number of male patients (data not shown). Table 2 summarizes results.

**Table 2:** Comparison of DAS28-SR and B-SR in the entire population

	DAS28-SR^a^	B-SR^a^	*p*
N° (% [95% CI]) of patients who achieved SR	78 (53.8 [46–62])	63 (43.4 [35–52])	0.000
Months of follow-up to SR^b^	9.1 ± 5	10.9 ± 5.6	0.023
Months of SR duration^b^	22.3 ± 5.7	17.5 ± 6.2	0.000

CI = Confidence interval. DAS28-SR = Sustained remission according to the Disease activity index (28 joints considered). B-SR = Boolean sustained remission. SR = Sustained remission

^a^Data presented as (mean ± SD) and number (percentage [95% CI])

^b^Data restricted to 50 patients who met bot definitions of SR.

Table 1 also compares baseline and cumulative characteristics between females and males patients with DAS28-SR and B-SR. At cohort inclusion, no differences were found but B-SR males were 10 years older than their female counterpart (45.6 ± 6.3 vs. 35.6 ± 12.2 years, *p* = 0.01); cumulative (up to SR) treatment and comorbidity were also similar but DAS28-SR males had higher number of comorbidities/patients than their female counterpart (1.5 ± 0.8 vs. 1.3 ± 0.8, *p* = 0.03). Data are summarized in Table 1.

### PRO in DAS-28 SR and B-SR patients

**Fig. 1:**
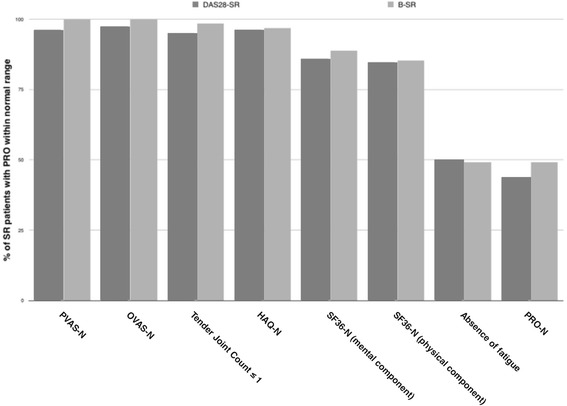
N° (%) of patients who achieved PRO norms. Figures depicts the number (bottom of the bars) and percentage of DAS28-SR patients (dark grey bar) and B-SR patients (light grey bar) who achieved PVAS, OVAS, HAQ and SF-36 norms, ≤ 1tender joints, absence of fatigue and all PRO norms (PRO-N)

The following (mean ± SD) PRO at either DAS28-SR or B-SR are summarized in Table 3 **:** PVAS, OVAS, tender joints count, HAQ, physical and mental component of the SF-36 and fatigue. In general, at SR, patients had PRO proxy to normal values. In addition, the percentage (95%CI) of patients with normal PRO varied from 97% (91–99) for HAQ-N, but decreased to 50% (39–61) for absence of fatigue, as shown in Fig. 1. There were no differences between females and males PRO scores at either DAS28-SR or B-SR (data not shown).

**Table 3:** (Mean ± SD) scores at DAS28-SR and B-SR

	DAS28-SR^a^ *N* = 78	B-SR^a^ *N* = 63
PVAS (0–100 mm)	2.5 ± 3.3	1.7 ± 2
OVAS (0–100 mm)	2.2 ± 3	1.6 ± 2.1
Tender joint count (0–68 joints)	0.2 ± 0.6	0.06 ± 0.3
HAQ (0–3)	0.04 ± 0.2	0.12 ± 0.8
Mental component of the SF-36 (0–100)	89.3 ± 11.5	91.1 ± 8.5
Physical component of the SF-36 (0–100)	86.9 ± 9.6	87.9 ± 8.9
Fatigue (0–100)	76.9 ± 15.6	76.4 ± 17.6

DAS28-SR = Sustained remission according to the Disease activity index (28 joints considered). B-SR = Boolean sustained remission. PVAS = Pain visual analogue scale. OVAS = Overall disease visual analogue scale. HAQ = Health assessment questionnaire. SF = Short-Form 36v2 Survey

^a^Data presented as (mean ± SD)

We compared the number (%) of patients who achieved PRO norms within the 50 patients who met DAS28-SR and B-SR, and no differences were noted. However, B-SR patients achieved a higher SF-36 mental component compared with their counterparts (92.3 ± 7.4 vs. 90.6 ± 0.1, *p* = 0.041).

### Months of follow-up to achieve outcome norms in patients with DAS28-SR and B-SR

**Fig. 2:**
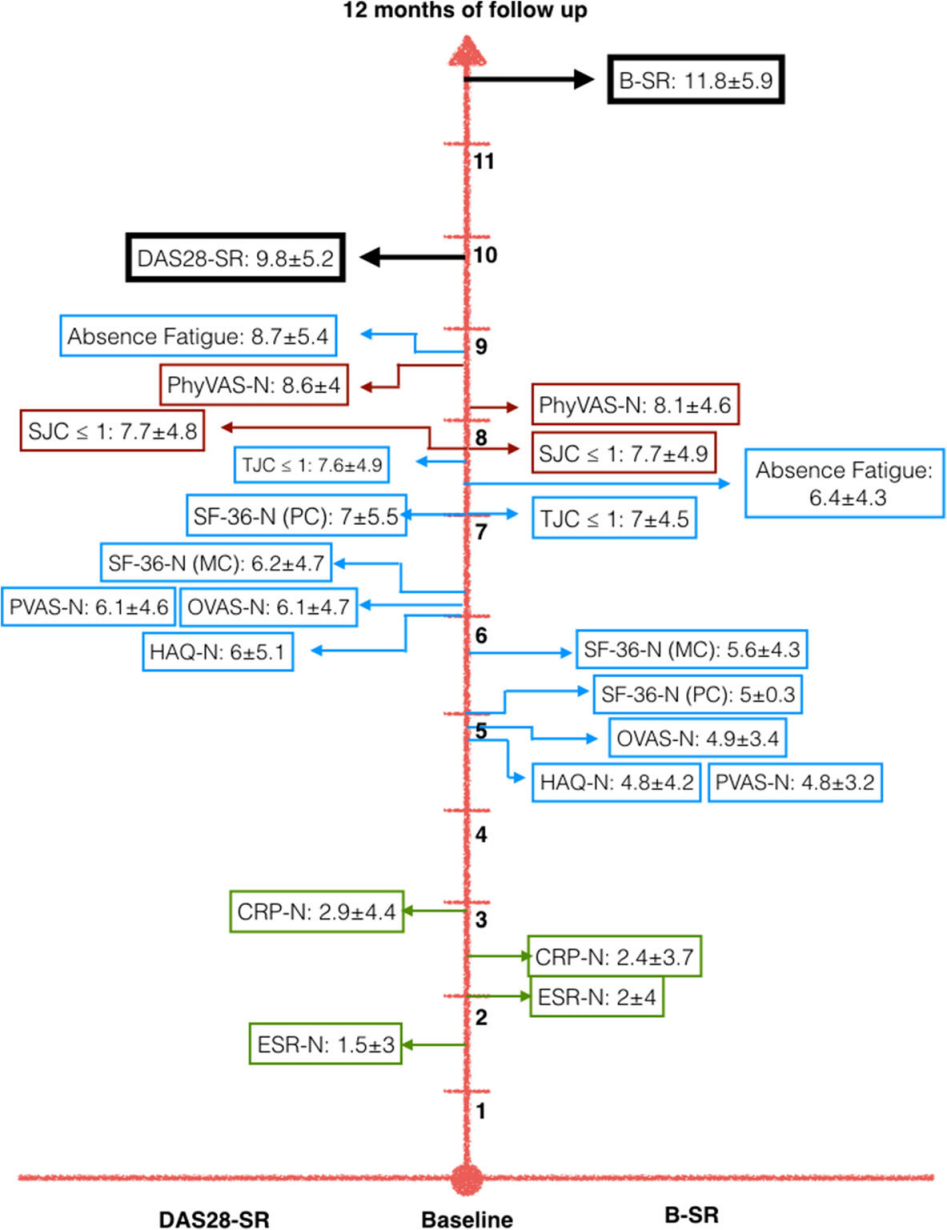
Months of follow-up to achieve outcomes norms in patients with DAS28-SR and B-SR

In the population of DAS28-SR patients, we calculated the (mean ± SD) months of follow-up to achieve the norm for each PRO; we also determined the time to achieve values within the normal range of ESR and CRP and the time to achieve ≤1 swollen joint and PhyVAS-N. The results from the entire population are summarized in Table 4. The number of patients with DAS28-SR who achieved the norm varied for each outcome, from 34 patients who referred absence of substantial fatigue to 77 patients with PVAS-N, PhyVAS-N and ESR-N. Interestingly, normalization of ESR and CRP was detected very early (at 1.5 and 2.9 months from baseline, respectively), followed by HAQ-N, PVAS-N, OVAS-N, SF-36-N (achieved between 6 and 7 months of follow-up), and tender joint count ≤1 (at 7.6 months). Absence of fatigue was detected late, at 8.7 months of follow-up, similar to DAS28-SR, which was achieved at (mean) 9.8 months (Fig. 2).

**Table 4:** (Mean ± SD) months of follow-up to outcome normalization in the 78 patients with DAS28-SR and the 63 patients with B-SR

Outcome, (N° of patients who achieved norm in DAS28-SR/B-SR patients)	Months of follow-up to outcome normalization in 78 DAS28-SR patients^a^	Months of follow-up to outcome normalization in 63 B-SR patients^a^
ESR-N, (77/61)	1.5 ± 3	2 ± 4
CRP-N, (73/63)	2.9 ± 4.4	2.4 ± 3.7
HAQ-N, (75/63)	6 ± 5.1	4.8 ± 4.2
PVAS-N, (77/63)	6.1 ± 4.6	4.8 ± 3.2
OVAS-N, (76/63)	6.1 ± 4.7	4.9 ± 3.4
SF-36-N, mental component, (67/54)	6.2 ± 4.7	5.6 ± 4.3
SF-36-N, physical component, (60/48)	7 ± 5.5	5 ± 3.3
Tender joint count ≤1(0–68 joints), (76/63)	7.6 ± 4.9	7 ± 4.5
Swollen joint count ≤1 (0–66 joints), (72/63)	7.7 ± 4.8	7.7 ± 4.9
Physician overall disease ≤10 (0-100 mm), (77/63)	8.6 ± 4.4	8.1 ± 4.6
Absence of substantial fatigue, (34/24)	8.7 ± 5.4	6.4 ± 4.3

N° = Number. DAS28-SR = Sustained remission according to the Disease activity index (28 joints considered). B-SR = Boolean sustained remission. ESR-N = Erythrocyte sedimentation rate within norms. CRP-N = C-reactive protein within norms. HAQ-N = Health assessment questionnaire within norms. P-VAS-N = Pain visual analogue scale within norms. O-VAS-N = Overall disease-visual analogue scale within norms. SF-36-N = Short-Form 36v2 survey within norms

^a^Data presented as (mean ± SD)

In the 63 patients with B-SR, a similar pattern was observed in the number of patients who achieved each particular outcome. In addition, normalization of the acute reactant phases was identified early during follow-up, followed by HAQ-N, PVAS-N, OVAS-N and both components of the SF-36. Of note, absence of fatigue was detected at 6.4 months of follow-up, before swollen and tender joint counts and PhyVAS-N (Table 4, Fig. 2).

Finally, we compared the follow-up to outcomes within the 50 patients who met both SR definitions. No differences were observed between both groups, but absence of substantial fatigue and physical component SF-36-N were achieved significantly earlier in B-SR patients (*p* ≤ 0.02).

## Discussion

Remission (sustained) has become the best clinical outcome in RA patients and may be approached as a state of no or minimal inflammatory disease activity associated with a halt of progression of joint damage [1], better PROs, working productivity and lower costs [24]. Today, it is a feasible target and the first recommendation from the “Treat-to-Target” approach [25]; nonetheless, patient’s perception of SR does not necessarily correspond to the physician construct of SR.

In the present study, we examined the impact of SR on PROs in a real clinical setting of an ongoing early arthritis clinic where completed, periodic and standardized assessments were performed by a dedicated rheumatologist. Two definitions of SR were used with a different degree of strictness [26] that allowed us to compare how each construct affected patient’s health related quality of life. The PRO examination included those adopted through consensus by the ACR, EULAR and Outcomes measures in Rheumatology (OMERACT) as well as fatigue, which was more recently added based on input from patient research partners [27, 28]. In addition, acute reactant-phase determinations and physician-derived measures were also included for additional (instead of a competitive) interpretation of the data.

We first found that SR was achieved in a substantial proportion of patients (i.e., 43% and 54%, depending on the definition used), and up to 35% met both definitions. In early disease, up to 60% of patients achieve clinical remission according to DAS28 [2, 29, 30]. We found a high percentage of B-SR and performed a 28-joint count, although it is recommended to include feet and ankle assessments when applying these criteria [4]. An extended joint count may favor the eventual detection of additional joints as either tender or swollen. Similar to previous publications, the highest percentage of SR patients was achieved with the DAS28 cut-off [8, 26, 29]. We additionally found that follow-up to first SR and time in SR favor DAS28-SR that was easier to achieve and maintain. DAS28 is considered to have lesser stringency than the Boolean definition of remission [29–31]. The more stringent DAS28 cutoff value (2.4 instead of 2.6), allowed the presence of up to 12 swollen joints while the ESR was restricted to practically normal values; in addition, it is possible to have up to 5 swollen joints and still being in remission using the DAS28 criteria if the other variables are nearly normal [29]. Finally, DAS28 was also less stringent with respect to elevation of the global assessments of activity and pain as compared to an other validated index, the Simplified Disease Activity Index (SDAI) [32].

Second, at SR, patients from our study had PRO proxy to normal values. Linde et al. [8] found that the health-related quality of life scores from Danish RA patients in clinical remission approached that of the general population. By contrast, Radner et al. [24] showed that patients in remission, on average, did not reach normal health-related quality of life values (from HAQ, SF-36 physical component, EURO Qol-5D and Short Form-6D). Important considerations to be highlighted included that their patients had almost 12 years of disease duration with the consequent accrual of irreversible damage and disability [33] and their population was almost 25 years older than ours. Physical function declines with age [34], and increased age reduces HRQoL in aged RA patients [35]. Finally, in our study, SR (instead of time point remission) was defined, and it has been shown that physical function (as per HAQ) continues to improve over time if remission is reached and sustained [36].

Third, the percentage of patients with normal PRO varied from 97% for the HAQ, but decreased to approximately 50% for absence of fatigue and did not differ according to the SR definition used. Fatigue is a symptom that patients consider to be as severe as pain, and they frequently experience fatigue as incontrollable and overwhelming [37]. Among patients who achieved remission (or low disease activity), fatigue is frequent and may be experienced as a problem that affects quality of life [38–40]. Additionally, a relationship between female sex and high levels of fatigue has been documented in a systematic review [41]. Our cohort was highly represented by females, which may explain the considerable prevalence of substantial fatigue in patients with SR, a number that was independent of the stringency of the SR definition used.

Fourth, we identified a particular temporal pattern of outcome normalization in patients who achieved SR, characterized by consecutive (rather than simultaneous) patient-centered followed by physician-centered, outcome normalization, with only mild differences between both SR definitions. Acute reactant phase normalization was detected very early during follow-up, followed by the simultaneous normalization of the majority of PRO, physician-dependent outcomes (as were swollen joint count and overall disease VAS) and SR construct. Moreover, 7 to 9 months extended between the normalization of acute reactant phases and the time point when the SR criteria are achieved. The results emphasize that the impact of SR on serologic disease activity, PRO and clinical disease activity do not necessarily occur in parallel, as previously highlighted by Altawil et al. [42]. In addition, SR according to DAS28 and the Boolean definition had a similar degree of stringency regarding their gain in most of the outcomes relevant to patients. In terms of fatigue and the physical component of the SF-36, it did behave similarly, but absence of fatigue and normalization of the physical component of the SF-36 were achieved earlier when the Boolean definition was used. The most strict definition translated into (2 specific) earlier improved patient-reported long-term outcomes. We do not have a clear explanation for the behavior of those outcomes. Nonetheless, Thiele et al. compared the functional ability of German RA patients in remission according to the DAS28, the SDAI and the Boolean definition of remission; they showed small differences between the 3 remission groups in swollen joint counts, moderate differences in acute phase reactants but large differences in PRO; patients achieving Boolean remission had a better patient global assessment as well as lower pain and fatigue scores than those in SDAI remission meanwhile for patients with DAS28 remission, disease duration, pain, fatigue and comorbid spine disease were associated with not achieving the other 2 sets of remission criteria [26].

The limitations of the study should be considered when interpreting our results. B and DAS-28 –SR’s definition include a patient VAS that can be high in patients with comorbid musculoskeletal conditions given that patients may not be able to distinguish between discomfort due to comorbid conditions and due to RA. Comorbid conditions were highly prevalent in our cohort [18]. We applied DAS28-SR using DAS28-ESR. An association between the ESR level and age [43] has been described, and elderly patients (3.2% of our cohort had ≥65 years of age) with true SR may have been misclassified as with some disease activity. Also, age (and gender) influence normative data of PROs (as SF-36 and HAQ); nonetheless, older age was underrepresented in our cohort and its impact on SR assessments is likely to be minimal. Fatigue was assessed using the fatigue short form 36 vitality subscale instead of a validated scale or an appropriated questionnaire primarily designed to assess fatigue [44]. Our cohort of patients is highly represented by females as previously described [17, 18] and data reported should not be generalized to RA males. Finally, additional confounding factors highly prevalent in RA patients [45] with confirmed strong associations with PRO, such as depression and anxiety, were not possible to control for given that these factors were not part of the original program in the patient’s follow-up.

## Conclusions

In conclusion, awareness of the patient preferences is a salient premise for priorities in health care as it has been demonstrated that patients and health professionals differ in their perceptions of patients’ health status and need for care [46, 47]. Remission status is the most desirable outcome. For adoption by both patients and physicians, remission status should reflect symptom’s resolution and ideally prevent subsequent structural damage. PRO provide unique information that cannot be collected from a physician, especially regarding symptoms, and complete the picture of patients who live with RA in the clinical context of sustained remission.

## Abbreviations

ACCP: Antibodies to cyclic citrullinated proteins
ACR: American Colleague of Rheumatology
B-SR: Boolean definition of sustained remission
CRP: C-reactive protein
DAS28: Disease activity score on 28 joints
DMARDs: Disease modifying anti-rheumatic drugs
EAC: Early Arthritis Clinic
ESR: Erythrocyte sedimentation rate
EULAR: European League Against Rheumatism
HAQ: Health assessment questionnaire
HAQ-N: Health assessment questionnaire- norm
HRQoL: Health-related quality of life
OMERACT: Outcomes measures in Rheumatology
O-VAS: Overall disease-visual analogue scale
OVAS-N: Overall disease-visual analogue scale-norm
PhyVAS-N: Physician overall disease visual analogue scale normalization
PRO: Patient-reported outcomes
P-VAS: Pain visual analogue scale
PVAS-N: Pain visual analogue scale-norm
RA: Rheumatoid arthritis
RF: Rheumatoid factor
SF-36-N: Short-Form 36v2 Survey-norm
SR: Sustained remission
SR-36: Short-Form 36v2 Survey
